# Barriers to the Access and Use of Rituximab in Patients with Non-Hodgkin’s Lymphoma and Chronic Lymphocytic Leukemia: A Physician Survey

**DOI:** 10.3390/ph7050530

**Published:** 2014-05-07

**Authors:** William H. Baer, Archana Maini, Ira Jacobs

**Affiliations:** 1 MercyHealth-ClinXus LLC, 260 Jefferson St., Grand Rapids, MI 49503, USA; 2 Broward Health Medical Center, 1625 SE 3rd Ave. #525, Ft. Lauderdale, FL 33314, USA; E-Mail: amaini@browardhealth.org; 3 Pfizer Inc., 235 East 42nd St., New York, NY 10017, USA; E-Mail: ira.jacobs@pfizer.com

**Keywords:** rituximab, biosimilar, access, emerging markets

## Abstract

Biologics such as rituximab are an important component of oncology treatment strategies, although access to such therapies is challenging in countries with limited resources. This study examined access to rituximab and identified potential barriers to its use in the United States, Mexico, Turkey, Russia, and Brazil. The study also examined whether availability of a biosimilar to rituximab would improve access to, and use of, rituximab. Overall, 450 hematologists and oncologists completed a survey examining their use of rituximab in patients with non-Hodgkin’s lymphoma (NHL) and chronic lymphocytic leukemia (CLL). Less than 40% of physicians considered rituximab as easy to access from a cost perspective. Furthermore, many physicians chose not to treat, were unable to treat, or had to modify treatment with rituximab despite guidelines recommending its use in NHL and CLL patients. Insurance coverage, reimbursement, and cost to patient were commonly reported as barriers to the use of rituximab. Across all markets, over half of physicians reported that they would increase use of rituximab if a biosimilar was available. We conclude that rituximab use would increase across all therapy types and markets if a biosimilar was available, although a biosimilar would have the greatest impact in Brazil, Mexico, and Russia.

## 1. Introduction

Rituximab, marketed under the trade names Rituxan™ (Genentech/Biogen Idec, South San Francisco, CA/Cambridge, MA, USA) in the United States (US) and MabThera™ (Roche, Basel, Switzerland) in the rest of the world, is a chimeric monoclonal antibody against the antigen CD20 [1,2,3]. CD20 is a transmembrane protein expressed in pre-B and mature B lymphocytes, and is involved in the activation process for cell cycle initiation and differentiation [4,5]. Binding of rituximab results in the death of CD20-expressing B lymphocytes through a variety of mechanisms [6,7]. Thus, rituximab is an important component of oncology treatment strategies and is commonly used to treat certain hematological malignancies [7,8,9]. It is indicated for the treatment of non-Hodgkin’s lymphoma (NHL), including follicular lymphoma (FL) and diffuse large B-cell lymphoma (DLBCL) subtypes, and chronic lymphocytic leukemia (CLL) in both the US and European Union [1,2]. Indeed, studies in patients with FL [10,11,12,13], DLBCL [14,15,16], and CLL [17,18] have shown that the addition of rituximab to standard therapy results in decreased disease progression and significantly improved survival rates. For example, the addition of rituximab to a treatment regimen of cyclophosphamide, doxorubicin, vincristine, and prednisone improved 10-year overall survival rates in patients with DLBCL from 27.6% to 43.5% [15]. Although not indicated, rituximab is also used for the treatment of CD20-expressing Hodgkin’s lymphoma [19], mantle cell lymphoma [20], Burkitt’s lymphoma [21], and Waldenstrom’s macroglobulinemia [22].

Biologics such as rituximab are structurally complex compounds developed from living organisms. This class of agents includes blood and plasma products, non-recombinant proteins isolated from natural sources, and recombinant proteins or antibodies produced in cell/tissue culture by way of recombinant DNA technology or controlled gene expression [23]. The development and manufacturing of biologics are complex and often expensive [3,24,25]. The high cost of biologic therapies like rituximab is a major barrier to oncologists and hematologists, particularly in countries with limited financial/healthcare resources. However, the development and use of biosimilars have the potential to increase the accessibility of targeted biologic therapies [3,25,26].

The term “biosimilar” refers to a biologic drug that is developed to be highly similar to an existing licensed reference biologic. Biosimilars are intended to treat the same diseases as the reference biologic using the same dose and treatment regimen. Unlike generic versions of chemically synthesized small molecule therapies, biosimilars are not structurally identical to their reference biologic. This is due to the purity, characteristics, and activity of a specific biologic being dependent on, and sensitive to, changes in the process by which it was manufactured [23,27]. Therefore, the aim is to create a product that is highly similar to a reference biologic, with no clinically meaningful differences between the biosimilar and the reference biologic in terms of safety, purity, and potency [23,25,26]. Biosimilars are commonly priced 25% to 30% less than the innovator biologic and increase access to specific therapies by offering patients a more affordable treatment alternative [25,28]. As biologic patents expire in the Unites States and European Union, the development and use of biosimilars is expected to grow substantially in the near future [3,25,26]. This was the impetus for the Biologics Price Competition and Innovation Act of 2009 (part of the Patient Protection and Affordable Care Act), which sets standards for the development, clinical testing, and approval of biosimilars in the US [29]. Similar standards have been established in Europe based on recommendations set by the European Medicines Agency Committee for Human Medicinal Products [30].

With these issues in mind, the objectives of the current study were to examine the true level of access that physicians in the US, Mexico, Turkey, Russia, and Brazil have to Rituxan™/MabThera™ and to identify potential barriers that may prevent the use of rituximab, as recommended by treatment guidelines [31,32,33], in patients with NHL or CLL. An additional objective of this study was to determine whether the introduction of a biosimilar to rituximab would improve access to, and use of, rituximab in these countries.

## 2. Experimental

### 2.1. Approach

A survey was designed to yield data on the access to, and usage of, rituximab by hematologists or oncologists in the US and emerging markets, including Brazil, Mexico, Russia, and Turkey. The survey was administered from July to August 2013.

### 2.2. Recruitment

Potential survey participants were identified and engaged by recruiters in each country. Recruiters were native speakers with insight into their country’s healthcare systems and cultures. Physicians were identified from proprietary databases, association lists, phone directories, and other commercially available sources. Alternatively, physicians who were already part of a recruiter’s online community could request to be part of the survey. Physicians were invited via phone or email and explained the purpose of the survey, why they were chosen to participate, and assured confidentiality of their responses. Those who agreed to participate were sent an email containing a link to the automated online survey. Physicians had to meet the following requirements, via a short screener survey, to participate in the main survey: full-time hematologist or medical oncologist; have been practicing for 2–35 years; ≥75% of time spent in direct patient care; practice is a mixture of hospital- and office-based; and ≥10% of their patients have CLL (at least 10 patients), NHL (at least 25 patients), or other malignancies. Upon completion of the main survey, participants were credited with a gift certificate or honorarium depending on country. Follow-up emails were sent to participants who began, but did not complete, the survey.

### 2.3. Survey Details

The survey asked questions related to patients with NHL and CLL. Specific questions were related to the following: type of malignancy; disease categories; types of patient insurance encountered; factors considered when making treatment decisions; treatment guidelines commonly followed; accessibility of rituximab; use of rituximab; barriers to the use of rituximab; and questions related to their potential use of a less-expensive (biosimilar) version of rituximab if it were available. Questions were answered in a variety of forms including: yes/no; percentages (example: what percentage of your patients are A, B, or C); 5- or 7-point Likert scales (examples: 1 = never to 5 = always; 1 = very easy to 7 = not at all easy); and select all that apply (example: A through Z). In certain cases, participants were given a text box to further explain their answers if necessary. The survey took approximately 10 minutes to complete.

### 2.4. Data

In total, 450 physicians completed the survey: 150 in the US and 75 each in Brazil (BRZ), Mexico (MEX), Russia (RUS), and Turkey (TUR). Data are presented as the percentage of physicians reporting a specific response and are given for physicians across all markets and in each individual market examined. The margins of error at a 90% confidence interval were ±3.9% (all markets combined), ±6.7% (US), and ±9.5% (BRZ, MEX, RUS, TUR). The margins of error at a 95% confidence interval were ±4.6% (all markets combined), ±8.0% (US), and ±11.3% (BRZ, MEX, RUS, TUR).

## 3. Results

### 3.1. Cancer Patient Population

Initially, physicians were asked to describe the cancer patient population under their care. Overall, physicians reported that roughly half (48%) of their cancer patients had a hematological malignancy as opposed to a solid malignancy (Table 1). Across all markets, NHL was the most commonly reported hematological malignancy (40% of all cases), whereas CLL accounted for 26% of all cases and “other hematological malignancies” accounted for the remaining 34% (Table 1). A majority of CLL cases (55% of all CLL patients) were classified as first-line therapy. Likewise, 56% of all FL patients and 60% of all DLBCL patients were classified as first-line therapy.

**Table 1:** Description of the cancer patient population.

Type of malignancy	Overall	US	BRZ	MEX	RUS	TUR
Solid malignancy, % of all cancer patients	52	58	44	56	43	53
Hematological malignancy, % of all cancer patients	48	42	56	44	57	47
**Type of hematological malignancy**						
NHL, % of hematological malignancy patients	40	37	44	46	36	43
Follicular lymphoma, % of NHL patients	45	55	46	38	43	36
Diffuse large B-cell lymphoma, % of NHL patients	55	45	55	62	57	64
CLL, % of hematological malignancy patients	26	24	33	24	28	20
Other, % of hematological malignancy patients	34	39	23	30	37	37

US = United States; BRZ = Brazil; MEX = Mexico; RUS = Russia; TUR = Turkey; NHL = non-Hodgkin’s lymphoma; CLL = chronic lymphocytic leukemia.

Across all markets, only 14% of physicians reported that their NHL and CLL patients differ with respect to types of insurance coverage utilized. Patients with NHL and CLL were most likely to receive government-funded insurance, particularly in Turkey and Russia (Overall = 51%, US = 39%, BRZ = 27%, MEX = 40%, RUS = 76%, TUR = 86%). Overall, 20% of patients received private medical insurance through their employer (US = 30%, BRZ = 36%, MEX = 15%, RUS = 2%, TUR = 6%), whereas 17% (US = 19%, BRZ = 27%, MEX = 27%, RUS = 7%, TUR = 6%) paid for their own private insurance. Patients in Mexico and Russia were the most likely to have no insurance and pay for medical expenses out of pocket (Overall = 9%, US = 5%, BRZ = 7%, MEX = 18%, RUS = 15%, TUR = 3%).

### 3.2. Factors Considered When Treating CLL and NHL Patients

Physicians were then asked to identify specific factors they consider when making decisions regarding the treatment of patients with NHL and CLL. Across all markets, physicians reported “goals of therapy” (76%), “treatment guidelines” (73%), and “patient co-morbidities” (73%) as the factors most considered. These were followed by “patient age” (67%), “recommendations from thought leaders” (50%), “patient insurance status” (32%), and “geographic locale” (16%).

A majority of all physicians (Overall = 60%) ranked the National Comprehensive Cancer Network (NCCN) guidelines as the most influential treatment guidelines with respect to NHL and CLL patients. Indeed, 71% (US = 92%, BRZ = 49%, MEX = 87%, RUS = 32%, TUR = 73%) of all physicians considered NCCN guidelines when treating patients. Guidelines from the American Society of Clinical Oncology (Overall = 45%, US = 43%, BRZ = 38%, MEX = 68%, RUS = 24%, TUR = 52%) and European Society for Medical Oncology (Overall = 38%, US = NA, BRZ = 21%, MEX = 53%, RUS = 26%, TUR = 55%) were also commonly taken into consideration by physicians. Local hospital guidelines were considered by 39% of physicians in Russia.

Physicians were then asked whether the specific clinical guidelines they follow, with respect to the treatment of CLL patients, explicitly recommend the use of rituximab. Across all markets, physicians reported that rituximab is more explicitly recommended for use as first-line therapy (Overall = 86%, US = 95%, BRZ = 93%, MEX = 85%, RUS = 75%, TUR = 75%) or in a relapsed setting (Overall = 82%, US = 89%, BRZ = 92%, MEX = 88%, RUS = 73%, TUR = 63%) than in a refractory setting (Overall = 58%, US = 60%, BRZ = 78%, MEX = 49%, RUS = 55%, TUR = 48%). Physicians were also asked whether the clinical guidelines they follow explicitly recommend the use of rituximab for the treatment of FL. Across all markets, physicians reported that rituximab is more explicitly recommended for use as first-line therapy (Overall = 91%, US = 95%, BRZ = 97%, MEX = 95%, RUS = 87%, TUR = 79%) and as first-line consolidation/maintenance therapy (Overall = 89%, US = 90%, BRZ = 96%, MEX = 88%, RUS = 88%, TUR = 80%) compared with second-line (Overall = 84%, US = 86%, BRZ = 87%, MEX = 83%, RUS = 87%, TUR = 75%) or second-line consolidation/maintenance therapy (Overall = 76%, US = 71%, BRZ = 86%, MEX = 75%, RUS = 84%, TUR = 68%). Finally, physicians were asked the same question with respect to DLBCL patients. Across all markets, physicians reported that rituximab is more explicitly recommended for use as first-line therapy (Overall = 93%, US = 95%, BRZ = 99%, MEX = 99%, RUS = 88%, TUR = 85%) as opposed to use in a relapsed or refractory setting (Overall = 81%, US = 80%, BRZ = 88%, MEX = 75%, RUS = 85%, TUR = 76%).

### 3.3. Access to Rituximab

Across all markets, only 39% of physicians considered rituximab easy to access from a cost perspective (Table 2). However, this was reported much more by physicians in the US and Turkey than by physicians in Brazil, Mexico, or Russia.

**Table 2:** Ease of access to rituximab (% of all physicians) ^a^.

Ease of access	*Overall*	US	BRZ	MEX	RUS	TUR
Easy to access ^b^	*39*	55	25	19	31	48
Middle ^c^	*54*	46	67	75	57	35
Not easy to access ^d^	*7*	0	7	7	12	17

^a^ Due to rounding, percentages may not add up to 100; ^b^ Corresponds to a score of 6 or 7 on a scale from 1 = not at all easy to access to 7 = very easy to access; ^c^ Corresponds to a score of 3, 4, or 5 on a scale from 1 = not at all easy to access to 7 = very easy to access; ^d^ Corresponds to a score of 1 or 2 on a scale from 1 = not at all easy to access to 7 = very easy to access; US = United States; BRZ = Brazil; MEX = Mexico; RUS = Russia; TUR = Turkey.

Among physicians who rated rituximab access in the middle (a rating of 3, 4, or 5 on a scale from 1 = not at all easy to access to 7 = very easy to access), issues related to cost were a primary reason, especially in Mexico and Russia (Overall = 37%, US = 31%, BRZ = 29%, MEX = 55%, RUS = 42%, TUR = 15%). Insurance coverage issues were also commonly reported, particularly outside the US (Overall = 22%, US = 9%, BRZ = 45%, MEX = 18%, RUS = 14%, TUR = 31%). Cost (Overall = 56%) and insurance (Overall = 31%) issues were also the most frequently reported reasons given by physicians who rated the access to rituximab as poor (a rating of 1 or 2).

### 3.4. Rituximab Usage and Barriers to Treatment

Physicians were asked to report how frequently (1 = always, 2 = frequently, 3 = not so often, 4 = rarely, 5 = never) they used rituximab in patients with CLL as a first-line therapy, in a relapsed setting, and in a refractory setting. Physicians who reported a score of 3, 4, or 5 were asked to identify the reasons they did not use rituximab in a certain setting (Table 3). Across all markets, common barriers to the use of rituximab as a first-line therapy or in a relapsed setting (the two settings in which rituximab use is most recommended by treatment guidelines) were related to treatment guidelines, cost to patients, accessibility to patients, and insurance coverage. Likewise, physicians were also asked to report how frequently they used rituximab in patients with FL. Across all markets, common barriers to the use of rituximab as a first-line therapy or as a first-line consolidation/maintenance therapy were related to: cost to patients, insurance coverage, and issue with patient compliance (Table 3). Finally, physicians were asked to report how frequently they used rituximab in patients with DLBCL. Across all markets, common barriers to the use of rituximab as a first-line therapy were related to: cost to patients, patient comorbidities, insurance coverage, efficacy, and accessibility to patients (Table 3).

All physicians were asked whether issues related to cost have affected their use of rituximab in the past. One third of all physicians, particularly those outside the US, commonly answered yes to the following question: “Are there any instances where you have not been able to treat a patient with rituximab or have had to delay their treatment due to reimbursement issues”, with percentages as follows: US = 16%, BRZ = 45%, MEX = 44%, RUS = 25%, TUR = 49%. Across all markets, the most common reasons for this inability or delay were related to insurance or payment issues (Table 4). Rituximab is typically administered to patients with NHL and CLL over 6–8 cycles [1,2]. A substantial percentage of physicians, particularly in Mexico, Russia and Turkey, reported that they have had to reduce the number of desired rituximab treatment cycles due to cost issues (Overall = 26%, US = 9%, BRZ = 17%, MEX = 52%, RUS = 30%, TUR = 41%). The three most common reasons for reducing the number of cycles were issues related to patient response, insurance coverage, and patients’ inability to pay (Table 4).

**Table 3:** Most common barriers to the use of rituximab (% of physicians) ^a,b^.

CLL Patients						
**As first-line therapy**	**Overall** (n = 64)	**US ** (n = 8)	**BRZ ** (n = 5)	**MEX ** (n = 11)	**RUS ** (n = 16)	**TUR** (n = 24)
Use not recommended by treatment guidelines or protocol I follow in this setting	20	25	0	27	19	21
High out-of-pocket treatment cost for patient	17	25	0	36	19	8
Not included in the formulary of drugs covered by patients’ insurance	16	0	40	9	6	25
**In relapsed setting**	**Overall ** (n = 80)	**US ** (n = 17)	**BRZ ** (n = 6)	**MEX ** (n = 9)	**RUS ** (n = 20)	**TUR ** (n = 28)
Use not recommended by treatment guidelines or protocol I follow in this setting	15	29	17	33	10	4
Not easily accessible for my patients	13	6	17	11	15	14
Patient compliance will be better with alternative treatment	11	12	0	0	15	14
Not included in the formulary of drugs covered by patients’ insurance	11	6	0	11	5	21
**In refractory setting**	**Overall ** (n = 188)	**US ** (n = 60)	**BRZ ** (n = 17)	**MEX ** (n = 38)	**RUS ** (n = 34)	**TUR ** (n = 39)
Not convinced of its efficacy in this setting	26	50	12	13	19	13
Use not supported by clinical trial data in this setting	12	15	18	8	18	3
Patient performance status/co-morbidities	12	7	18	16	3	21
**FL Patients**						
**As first-line therapy**	**Overall ** (n = 55)	**US ** (n = 7)	**BRZ ** (n = 2)	**MEX ** (n = 6)	**RUS ** (n = 15)	**TUR ** (n = 25)
High out-of-pocket treatment cost for patient	20	29	0	17	33	12
Patient performance status/co-morbidities	16	29	0	17	27	8
Not included in the formulary of drugs covered by patients’ insurance	13	0	50	0	0	24
Use not recommended by treatment guidelines or protocol I follow in this setting	11	14	0	17	20	4
Patient compliance will be better with alternative treatment	11	0	0	17	7	16
**As first-line consolidation/maintenance therapy**	**Overall ** (n = 73)	**US ** (n = 15)	**BRZ ** (n = 5)	**MEX ** (n = 10)	**RUS ** (n = 20)	**TUR ** (n = 23)
High out-of-pocket treatment cost for patient	16	13	0	10	38	9
Not included in the formulary of drugs covered by patients’ insurance	15	0	40	10	5	30
Not convinced of its efficacy in this setting	11	27	0	0	5	13
Patient compliance will be better with alternative treatment	10	13	20	10	0	13
**As second-line therapy and subsequent therapy**	**Overall ** (n = 98)	**US ** (n = 23)	**BRZ ** (n = 7)	**MEX ** (n = 23)	**RUS ** (n = 19)	**TUR ** (n = 26)
High out-of-pocket treatment cost for patient	13	9	0	13	26	12
Not included in the formulary of drugs covered by patients’ insurance	12	0	14	13	5	27
Patient performance status/co-morbidities	12	22	14	4	5	15
**As second-line consolidation/maintenance therapy**	**Overall ** (n = 135)	**US ** (n = 43)	**BRZ ** (n = 13)	**MEX ** (n = 28)	**RUS ** (n = 20)	**TUR ** (n = 31)
Not convinced of efficacy in this setting	17	26	31	14	5	10
Use not supported by clinical trial data in this setting	13	21	15	14	10	3
Patient performance status/co-morbidities	13	12	15	7	10	19
**DLBCL Patients**						
**As first-line therapy**	**Overall ** (n = 54)	**US ** (n = 11)	**BRZ ** (n = 2)	**MEX ** (n = 5)	**RUS ** (n = 16)	**TUR ** (n = 20)
High out-of-pocket treatment cost for patient	19	18	0	0	31	15
Patient performance status/co-morbidities	13	9	50	0	19	10
Not included in the formulary of drugs covered by patients’ insurance	11	0	50	20	0	20
Not convinced of efficacy in this setting	11	27	0	0	6	10
Not easily accessible for my patients	11	9	0	0	19	10
**In relapsed/refractory setting**	**Overall ** (n = 98)	**US ** (n = 28)	**BRZ ** (n = 9)	**MEX ** (n = 17)	**RUS ** (n = 19)	**TUR ** (n = 25)
High out-of-pocket treatment cost for patient	15	11	11	6	37	12
Not convinced of efficacy in this setting	15	32	11	12	5	8
Not included in the formulary of drugs covered by patients’ insurance	13	4	0	24	5	28

^a^ Percentages based only on those physicians who reported that they not so often, rarely, or never use rituximab. See “n” in each column for the number of physicians used to calculate each percentage; ^b^ Barriers reported by >10% of these physicians across all markets; CLL = chronic lymphocytic leukemia; FL = follicular lymphoma; DLBCL = diffuse large B-cell lymphoma; US = United States; BRZ = Brazil; MEX = Mexico; RUS = Russia; TUR = Turkey.

Across all markets, over half of physicians reported that they would increase their use of rituximab if a less-expensive (30% lower cost) version were available. This was especially evident in Brazil, Mexico, and Russia (Overall = 52%, US = 26%, BRZ = 77%, MEX = 76%, RUS = 63%, TUR = 47%).

**Table 4:** Most common reasons physicians had to cancel, delay, or reduce treatment with rituximab (% of physicians) ^a^.

Reasons for cancel or delay	**Overall** (n = 147)	**US ** (n = 24)	**BRZ** (n = 34)	**MEX** (n = 33)	**RUS ** (n = 29)	**TUR** (n = 37)
Insurance/government refused to fund the treatment	36	21	47	6	11	76
Patient had no insurance/not eligible for reimbursement	29	33	18	67	16	8
Patient unable to pay copayment	26	33	27	12	63	14
Hospital did not have funds to provide rituximab	8	4	9	12	11	3
**Reasons for cycle reduction**	**Overall** (n = 119)	**US** (n = 13)	**BRZ** (n = 13)	**MEX** (n = 39)	**RUS** (n = 23)	**TUR** (n = 31)
Patient response	31	15	31	54	26	13
Insurance coverage	24	62	31	15	13	26
Patient unable to pay	16	0	8	28	26	3
Hospital environment	9	0	0	0	0	36
Availability/lack of dugs	6	0	15	8	9	0
Issues with financing	4	0	0	3	17	0

^a^ Percentages based only on those physicians who reported having to cancel, delay, or reduce treatment with rituximab. See “n” in each column for the number of physicians used to calculate each percentage; US = United States; BRZ = Brazil; MEX = Mexico; RUS = Russia; TUR = Turkey.

## 4. Discussion

Physicians participating in this survey reported that roughly half of the cancer patients under their care were treated for a hematological malignancy (range across all markets = 42%–57%). NHL patients represented 40% of hematological malignancy cases, while CLL and “other” represented 26% and 34%, respectively. More than half of all patients with NHL or CLL were classified as first-line therapy. 

Nearly three fourths of all physicians reported that they consider clinical guidelines when making treatment decisions regarding their patients with NHL and CLL. More than 80% of physicians reported that the specific treatment guidelines they follow explicitly recommend the use of rituximab for the treatment of CLL (as a first-line therapy or in a relapsed setting), FL (as a first-line therapy, as a first-line consolidation/maintenance therapy, or as a second-line therapy), and DLBCL (as a first-line therapy or in a relapsed/refractory setting). Despite these treatment guidelines, a number of physicians across all markets (range = 12%–22%) reported that they “not so often”, “rarely”, or “never” use rituximab to treat their patients with CLL (as a first-line therapy or in a relapsed setting), FL (as a first-line therapy, as a first-line consolidation/maintenance therapy, or as a second-line therapy), or DLBCL (as a first-line therapy or in a relapsed/refractory setting). The most common reasons for not utilizing rituximab, across all markets and types of malignancy, were related to insurance coverage, cost to the patient, treatment guidelines, and patient comorbidities. Indeed, less than 40% of physicians considered rituximab as easy to access from a cost perspective. This was especially evident in Brazil (25%), Mexico (19%), and Russia (31%). Outside the US, issues related to cost and insurance coverage were most frequently cited by physicians rating access to rituximab ≤ 5 (on a scale from 1 = not at all easy to 7 = very easy). Such issues commonly led to a change in desired treatment strategy. One-third of all physicians, particularly those in Brazil (45%), Mexico (44%), and Turkey (49%) reported having had to delay treatment with rituximab due to reimbursement issues, mostly related to insurance or patient payment issues. Likewise, a substantial percentage of physicians, particularly in Mexico (52%), Russia (30%), and Turkey (41%) reported that they have had to reduce the number of desired rituximab treatment cycles, most commonly due to issues regarding patient response, insurance coverage, and patient’s inability to pay.

Overall, these data demonstrate that a population of physicians chooses not to treat, is unable to treat, or has to modify treatment with rituximab despite explicit guidelines recommending its use in patients with NHL and CLL. Although issues directly related to the patient (such as patient comorbidities) are part of this decision-making process, issues related to insurance coverage, reimbursement, and cost to the patient were commonly reported as barriers to the use of rituximab. Indeed, across all markets, over half of physicians reported that they would increase their use of rituximab if a less expensive version was available. Therefore, we conclude that rituximab use would increase across all therapy types and markets if a biosimilar was available. However, a biosimilar to rituximab would have the greatest impact in the Brazil, Mexico, and Russia since, of all markets examined, physicians from these countries were least likely to rate rituximab as easy to access from a cost perspective. In addition, patients from these countries, especially Mexico and Russia, were most likely to have to pay for medical expenses out of pocket due to a lack of insurance coverage. Finally, physicians from these countries were also the most likely to increase use of rituximab if a less costly version was available.

Such conclusions are reinforced by examining the introduction of Reditux™ to India. Reditux™ (Dr Reddy’s) was the first intended biosimilar developed for rituximab [34]. In 2007, Reditux was launched at 50% lower cost than the originator (MabThera™) [34]. Within three years of launch, the number of patients receiving rituximab therapy increased six-fold in India, where the gross national income *per capita* is roughly $1,500 (data for year 2012) [35] and where a majority of patients have to pay for a substantial portion of their own medical care [34]. Furthermore, a study comparing the cost of intended biosimilar *versus* originator versions of paclitaxel, gemcitabine, docetaxel, oxaliplatin, and irinotecan in India found savings of $900–$4300 associated with use of the intended biosimilar versions [36]. This resulted in yearly savings of approximately $843 million dollars for health systems in India [36]. Due to such cost savings, the use of biosimilars to rituximab is expected to substantially increase globally in the near future. Reditux™, for example, has now been marketed in Bolivia, Chile, and Peru [3]. In addition, Probiomed has marketed Kikuzubam™, another intended biosimilar to rituximab, in Mexico. Patents are set to expire shortly in both the US and European Union, providing more opportunities for the use of biosimilars to rituximab [3]. Indeed, a majority of oncologists surveyed (number surveyed = 183) from the US and Europe expect to prescribe biosimilars to common monoclonal antibody treatments within the first 12 months of their launch [37]. 

The availability of a biosimilar to rituximab will also benefit patient populations outside of the oncology arena. In the US and European Union, for example, rituximab is also approved for the treatment of refractory rheumatoid arthritis (in combination with methotrexate), and for the treatment of Wegener’s granulomatosis and microscopic polyangiitis (in combination with glucocorticoids) [1,2]. Rituximab is also used to treat idiopathic thrombocytopenic purpura [38], autoimmune hemolytic anemia [38], thrombotic thrombocytopenic purpura [39], and Evan’s syndrome [38]. Although rituximab is not FDA-approved for any of these benign hematological conditions, its use is supported by evidence-based medicine and recommended by major hematological treatment guidelines such as those provided by the American Society of Hematology. However, our research did not specifically address the use of rituximab in these conditions, and care must be taken when attempting to generalize our findings to other physician/patient populations.

Some of the findings presented in this study, as for all survey research, may be limited in that respondents may not precisely recall their experiences, particularly experiences that happened in the distant past. It should be noted that some survey questions are limited in that answers are framed in the “yes/no”, Likert scale, or multiple-choice formats, which may not capture detailed accounts of physician experience or additional, unanticipated responses. However, all attempts were made to include an option of “other: please explain” when appropriate in this survey. Finally, these surveys were conducted across multiple countries having different healthcare systems and cultural backgrounds. Care must be taken in attempting to generalize these findings to physicians from countries not included in the current study.

Our study did not specifically address factors taken into account by physicians when they are considering the use of a biosimilar. Is the safety and efficacy profile of a biosimilar to rituximab a potential concern for physicians? We may infer, at least among physicians who reported that they would use a biosimilar to rituximab, that the safety and efficacy of a biosimilar is not a barrier to use provided these parameters are sufficiently demonstrated in controlled clinical trials. Future research is warranted to determine precisely what type and amount of clinical data physicians examine before they are convinced of a biosimilar’s safety and efficacy. Additional data will also identify additional factors, other than safety and efficacy, that affect a physician’s decision to prescribe a biosimilar. Cost will certainly play a role in this decision. However, government regulations and payer recommendations greatly affect market access of biosimilars and, thus, influence physician treatment decisions. 

## 5. Conclusions

Overall, we conclude that a biosimilar to rituximab would increase the use of rituximab in the oncology arena, particularly in the emerging markets of Russia, Mexico, and Brazil. A biosimilar to rituximab would address an unmet need in patients with CLL and NHL who currently do not receive rituximab, or receive less than optimal treatment with rituximab, due to insurance coverage or cost issues related to such treatment.
